# The influences of stomatal size and density on rice abiotic stress resilience

**DOI:** 10.1111/nph.18704

**Published:** 2023-01-11

**Authors:** Robert S. Caine, Emily L. Harrison, Jen Sloan, Paulina M. Flis, Sina Fischer, Muhammad S. Khan, Phuoc Trong Nguyen, Lang Thi Nguyen, Julie E. Gray, Holly Croft

**Affiliations:** ^1^ Plants, Photosynthesis and Soil, School of Biosciences University of Sheffield S10 2TN Sheffield UK; ^2^ Institute for Sustainable Food, School of Biosciences University of Sheffield Sheffield S10 2TN UK; ^3^ Future Food Beacon of Excellence and the School of Biosciences University of Nottingham Nottingham NG7 2RD UK; ^4^ High Agricultural Technology Research Institute G9‐11, Street 31, Area 586, Phu Thu Ward, Cai Rang District Can Tho City Vietnam

**Keywords:** climate change, drought, plant water‐use, rice, salinity, stomata, temperature, vapour pressure deficit (VPD)

## Abstract

- A warming climate coupled with reductions in water availability and rising salinity are increasingly affecting rice (*Oryza sativa*) yields. Elevated temperatures combined with vapour pressure deficit (VPD) rises are causing stomatal closure, further reducing plant productivity and cooling. It is unclear what stomatal size (SS) and stomatal density (SD) will best suit all these environmental extremes.
- To understand how stomatal differences contribute to rice abiotic stress resilience, we screened the stomatal characteristics of 72 traditionally bred varieties. We found significant variation in SS, SD and calculated anatomical maximal stomatal conductance (*g*_smax_) but did not identify any varieties with SD and *g*_smax_ as low as transgenic *OsEPF1oe* plants.
- Traditionally bred varieties with high SD and small SS (resulting in higher *g*_smax_) typically had lower biomasses, and these plants were more resilient to drought than low SD and large SS plants, which were physically larger. None of the varieties assessed were as resilient to drought or salinity as low SD *OsEPF1oe* transgenic plants. High SD and small SS rice displayed faster stomatal closure during increasing temperature and VPD, but photosynthesis and plant cooling were reduced.
- Compromises will be required when choosing rice SS and SD to tackle multiple future environmental stresses.

## Introduction

Developing high‐yielding rice varieties that use less water which can withstand multiple abiotic stresses will be critical for maintaining future global food security (Singh *et al*., 2021). Currently, rice is the most consumed human food crop, providing > 20% of the total calories consumed worldwide (Fukagawa & Ziska, 2019). It takes *c*. 2500 l of water to produce 1 kg of grain, equating to *c*. 30% of the world's fresh water supplies being used for rice production (Bouman, 2009). Half of all rice cultivation is irrigated, which boosts crop yields and protects against drought and heat stress, but such practices are highly water‐intensive and often lead to anaerobic soils and excessive methane production (Singh *et al*., 2021). Most other farmed rice is rain fed, with 34% grown in lowland ecosystems, and 9% in upland environments. With less available clean water for irrigation, and a need to reduce emissions, dependence on rain‐fed rice agriculture will increase (Bouman, 2009; Singh *et al*., 2021). Such changes in farming practices are forecast to occur at the same time as severe droughts, higher temperatures, higher vapour pressure deficit (VPD) and rising soil salinity are all predicted to increase (Livsey *et al*., 2019; IPCC, 2021). Taken together, these factors have the potential to greatly impact global rice yields at a time when demand for rice (and clean water) is rapidly growing (Panda *et al*., 2021; Singh *et al*., 2021).

To regulate gaseous exchanges with the aerial environment, plants use microscopic epidermal valves called stomata (Zeiger *et al*., 1987). Individually termed a stoma or stomate, each valve contains a pair of guard cells that are adjustable via changes to internal turgor pressure. Increasing turgor promotes guard cell expansion and stomatal pore opening, whereas decreased turgor leads to guard cell relaxation and pore closing. These movements regulate carbon dioxide (CO_2_) entry for photosynthesis (*A*), and the release of water, termed stomatal conductance to water vapour (*g*_sw_). Increases in *g*_sw_ are often associated with increased plant transpiration (*E*), and when *E* rises, more water (and nutrients) are pulled up by roots from underlying soils (Matimati *et al*., 2014). A larger *E* (often aided via increased stomatal opening) promotes evaporative cooling when temperatures rise. This response enables *A* to be maintained and prevents leaves from overheating, but such responses are VPD dependent and can vary considerably between plant species (Urban *et al*., 2017; Caine *et al*., 2019; Durand *et al*., 2019; Ye *et al*., 2020). During drought, high water salinity and/or rising VPD, stomata often close, and this leads to reduced *g*_sw_ and *E*, which can restrict *A*, nutrient uptake, plant cooling and ultimately growth and seed yield (Merilo *et al*., 2018; Grossiord *et al*., 2020; Ma *et al*., 2020). VPD rises occur when the difference between the maximum amount of water the air can hold, and the actual amount of water in the air increases. This is often the case as temperatures rise (Grossiord *et al*., 2020), and forecasts predict that both temperature and VPD will continue to rise until the end of the century (Genua‐Olmedo *et al*., 2016; Yuan *et al*., 2019; IPCC, 2021). Taken together, the environmental factors mentioned here represent a contrasting set of demands for rice and their stomata, particularly as climate change intensifies.

With prolonged changes in environmental stimuli, many plant species alter stomatal development by adjusting stomatal size (SS) and/or stomatal density (SD) – often in opposite directions (Franks & Beerling, 2009; Franks *et al*., 2012). This developmental adjustment has been widely observed in living plants, herbarium samples and even fossil records, and also coincides with CO_2_ fluctuations that have occurred during different geological epochs. Typically, high CO_2_ environments are associated with an increased SS and reduced SD, and low CO_2_ environments are associated with the opposite conformation of SS and SD (Franks & Beerling, 2009). Such developmental adjustments have been suggested to alter operating *g*_sw_ range, owing to alterations in calculated anatomical maximum stomatal conductance (*g*_smax_), with high SD, small SS plants potentially able to achieve high operating *g*_sw_ because of a higher *g*_smax_ (Franks & Beerling, 2009; Bertolino *et al*., 2019). Alterations to SS and SD have also been shown to adjust stomatal response time with evidence suggesting that plants with smaller SS (often with higher SD) being more capable of rapidly responding to environmental conditions, and this has the potential to boost water‐use efficiency (Drake *et al*., 2013; McAusland *et al*., 2016; Bertolino *et al*., 2019; Durand *et al*., 2019). This responsiveness is suggested to occur as smaller stomata have greater guard cell membrane surface area to volume ratios which enables more rapid changes in solutes comparatively to larger stomata. For fluctuating light responses, this means faster responsivity of *g*_sw_, reducing unnecessary water loss (Lawson & Vialet‐Chabrand, 2019). Negative correlations between SS and speed have been observed within a species (or closely related species) but not necessarily between distantly related species (McAusland *et al*., 2016). However, other studies have shown that stomatal responsiveness is not always related to SS (Eyland *et al*., 2021), and the improved benefits of small SS on optimising *A* may be light dependent (Zhang *et al*., 2019). In monocot grasses such as rice, each stomatal guard cell pair is surrounded by a pair of subsidiary cells, which increase the speed of stomatal opening and closure (Franks & Farquhar, 2007; Raissig *et al*., 2017; Gray *et al*., 2020).

It is possible to improve abiotic stress resilience by genetically manipulating stomatal physiology or stomatal development (Huang *et al*., 2009; Mohammed *et al*., 2019). Previous work manipulating the levels of epidermal patterning factor (EPF) signalling peptides in rice and wheat (*Triticum aestivum* L.) has shown that large SD reductions of *c*. 58–80% can lead to significantly lower *g*_sw_ without significantly impacting *A* (Caine *et al*., 2019; Dunn *et al*., 2019; Mohammed *et al*., 2019). These larger reductions in *g*_sw_ compared to *A* led to improved intrinsic water‐use efficiency (iWUE) without negatively impacting seed yield. In fact, moderate reductions in SD, improved rice yields following drought imposition during the flowering stage (Caine *et al*., 2019). Surprisingly, EPF‐driven reductions in SD had inconsistent effects on SS between different rice varieties despite sampling at the same development stage (Caine *et al*., 2019; Mohammed *et al*., 2019). Overexpression of *OsEPF1* in transgenic IR‐64 plants resulted in smaller stomata (Caine *et al*., 2019), whereas *OsEPF1oe* Nipponbare plants had larger stomata (Mohammed *et al*., 2019).

It is now clear that reductions in SD can result in reduced crop water loss leading to better drought avoidance (Caine *et al*., 2019; Mohammed *et al*., 2019). In this study, we investigate whether selecting for specific SS and SD traits in rice could mitigate against not only drought, but also additional climate‐change‐associated abiotic stresses including rising salinity, temperature and temperature‐associated VPD. Specifically, we ask the following: (1) Is it possible to identify the combination of SS and SD found in IR‐64 *OsEPF1oe* plants in other traditionally bred high‐yielding rice varieties? (2) Do naturally bred varieties or *OsEPF1oe* plants with fewer, smaller stomata perform better during drought or exposure to high salinity? And (3) how does rice SS and SD affect responsiveness to rising temperature and associated leaf VPD.

## Materials and Methods

### Plant materials

A collection of 72 rice (*Oryza sativa* L.) varieties previously assayed for salinity tolerance were kindly provided by Jose De Vega, Earlham Institute (Supporting Information Table S1). Two independently transformed lines of IR‐64 variety overexpressing *OsEPF1* have been previously described (Caine *et al*., 2019).

### Plant growth conditions

Rice seeds placed in 15–20 ml of reverse osmosis (RO) water were sealed in Petri dishes with micropore tape (3 M; St Paul, MN, USA) and germinated under 12 h : 12 h, 26°C : 24°C, light : dark cycle at photosynthetically active radiation (PAR) 200 μmol m^−2^ s^−1^. Seedlings were transferred onto a previously described soil mix (Caine *et al*., 2019) in 0.8 l pots (IPP, Bytom, Poland). Pots were prepared by first half filling with soil mix, then RO water was mixed through, and then a second equal application of soil was added, and further RO water mixed through to saturate the soil. When drained, the soil level was *c*. 1.5 cm from the pot apex. For preparation of salt‐treated pots comparing IR‐64 with *OsEPF1oe* plants, a 20 mM solution of NaCl rather than RO water was applied to saturate the soil during mixing. For salinity tolerance experiments comparing nine naturally bred varieties with *OsEPF1oe* plants, all samples were initially transplanted into RO‐mixed soil, with 50 mM NaCl being first applied 8 d after transferring. This was at 16 d post germination (DPG). All seedlings were grown under 12 h : 12 h, 30°C : 24°C, light : dark, 60% relative humidity (RH) with CO_2_ concentration between 450 and 480 ppm. For initial stomatal screening and for salinity experiments comparing IR‐64 with *OsEPF1*oe plants, samples were grown in PGR‐15 Conviron (Controlled Environments Ltd, Winnipeg, MB, Canada) growth cabinets set 1000 μmol m^−2^ s^−1^ PAR at canopy level. For all other experiments, plants were grown in a Conviron BDW 160 cabinet set to 1500 μmol m^−2^ s^−1^ PAR at canopy level. A constant supply of RO water or salt water was available during experiments.

### Drought and salinity experiments

For droughted plants (*n* = 10 or 11), water was withheld for 5 d from 30–35 DPG. New leaves were classified as visible new growth emerging from sheaves of pre‐existing tillers at 42 DPG. For salinity experiments, salt water was applied when required, and trays changed weekly for 20 mM NaCl experiments (*n* = 6), or fortnightly for the 50 mM NaCl experiment (*n* = 7–9). In both cases, salt water or fresh water (for controls) was applied from above every time the salt water in trays was changed. Leaf blade and tiller base (2 cm from soil) Φ PSII measurements were collected 4–5 h into the photoperiod using a FluorPen FP 110 (PSI, Drasov, Czech Republic). Leaf discs were collected from 35 DPG fresh water and salt grown plants and dried in a 40°C oven for 3 d before analysis.

### Gas exchange and thermal imaging measurements

**Fig. 1:**
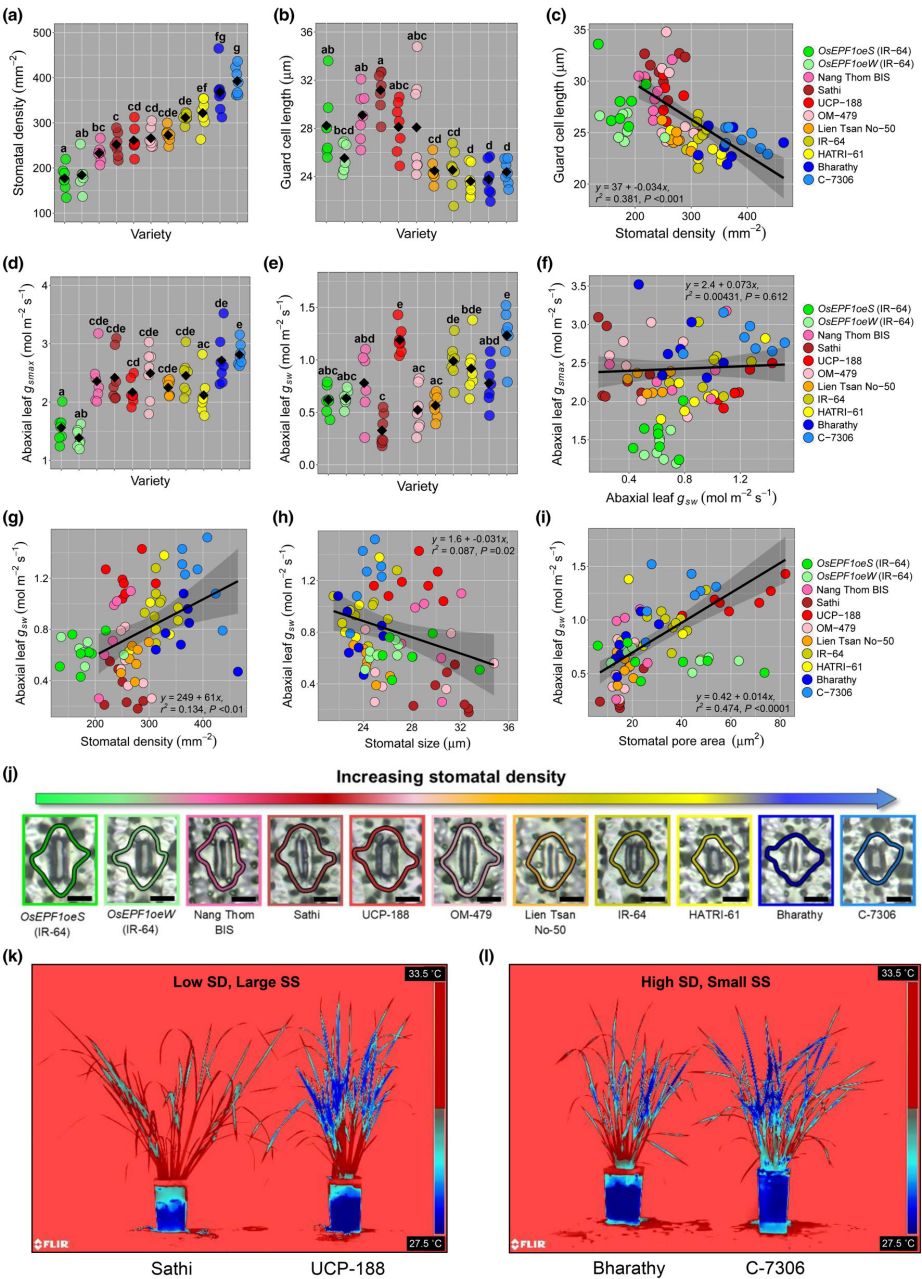
Stomatal size (SS), stomatal density (SD) and pore aperture contributions to rice (*Oryza sativa*) gaseous exchange on plants grown in high light conditions (1500 μmol m^−2^ s^−1^ PAR). (a) Abaxial SD and (b) SS (guard cell length) of nine selected rice varieties and two transgenic *OsEPF1oe* plants on the abaxial leaf surface. (c) Regression analysis between SS and SD. (d) Calculated maximum anatomical stomatal conductance (*g*_smax_), and (e) corresponding operating stomatal conductance (*g*_sw_). Regression analysis of (f) abaxial *g*_smax_ and abaxial operating *g*_sw_, (g) abaxial operating *g*_sw_ and SD, (h) abaxial operating *g*_sw_ and SS and (i) abaxial operating *g*_sw_ and stomatal pore area. (j) Examples of stomatal openness (Bar, 10 μm). (k) Thermal images for plants with low SD and large SS and (l) high SD and small SS. Different letters on graphs indicate a significant difference between the means (one‐way ANOVA, Tukey HSD test, *P* < 0.05). Black diamonds represent means. *OsEPF1oe* plants are excluded from regression analyses. (a–i) *n* = 7 plants.

Operating *g*_sw_ measurements were collected between 3 and 5 h into the photoperiod using a LI‐600 porometer (Li‐Cor, Lincoln, OR, USA) set to a flow rate of 150 μmol s^−1^ (*n* = 7). Thermal images in Fig. 1 were captured using a FLIR T650sc (Wilsonville, OR, USA). Steady state and dynamic gas exchange experiments were performed using LI‐6800 Portable Photosynthesis Systems (Li‐Cor) and attached Multiphase Flash Fluorometer (6800‐01A). Fully expanded leaves of 19–25 DPG (leaf 5 or 6) were used for steady‐state measurements of plants grown in 20 mM NaCl. Leaf chamber conditions were set to light intensity 2000 μmol m^−2^ s^−1^ PAR, 60% RH, *T*_air_ 30°C, flow 300 μmol s^−1^ and [CO_2_]_ref_ 480 ppm. Over a 5‐min period, 10 readings were taken and then averaged (*n* = 6 plants). *T*_leaf_ ranged from 31.50 to 32.42°C. For temperature and associated VPD incline experiments, fully expanded leaf 5 of 19–23 DPG plants were measured, with the leaf chamber set to 2200 μmol m^−2^ s^−1^ PAR, 55% RH, *T*_air_ 32°C, flow 400 μmol s^−1^ and [CO_2_]_ref_ 450 ppm. Once steady‐state was reached, two readings were taken at the end of 6‐min intervals, then the temperature was increased by 2.5°C in 4 × 6‐min intervals, with readings taken immediately before each subsequent temperature increase. Four further readings were recorded at 42°C (*n* = 7 or 8 plants). Leaf chamber matching was conducted before each reading and the *T*_leaf_ ranged from 31.26 to 33.20°C at *T*_air_ 32°C, 33.62 to 35.53°C at *T*_air_ 34.5°C, 35.71 to 37.88°C at *T*_air_ 37°C, 37.88 to 40.42°C at *T*_air_ 39.5°C and 39.79 to 43.06 at *T*_air_ 42°C. Steady‐state high temperature settings were the same as for the incline experiments, except *T*_air_ was 39°C and RH maintained at 55% throughout (*n* = 5 or 6). This temperature was chosen for steady‐state as it was the maximum temperature where RH could be maintained at the set point. The *T*_leaf_ range was from 37.72 to 39.83°C.

### Analysis and quantification of stomatal traits

Epidermal imaging and quantification was conducted on Imagej using nail varnish impressions of dental resin imprints taken from the abaxial side of leaf 5 (19–23 DPG), or leaf 8 (28–33 DPG). Two 0.44 mm^2^ fields of view per replicate were used to calculate SD with 5 stomata per biological replicate used to assess guard cell length.

Calculations for abaxial anatomical *g*_smax_ were performed based on double end‐corrected version of the Franks & Farquhar (2001) equation from Dow *et al*. (2014) where *d* (m^2^ s^−1^) is the diffusivity of water in air and *v* (m^3^ mol^−1^) is the molar volume of air. Maximum pore aperture *a*_max_ (μm^2^) was calculated as an ellipse from axes equal to the measured aperture length and half of the aperture length. Pore depth *l* (μm) was taken as equal to guard cell width at the centre of the stoma and *D* (mm^−2^) is SD.

$$\;\text{Abaxial anatomical}\;g_{\text{smax}}=\left(d\cdot D\cdot a_{\max}\right)/\left(v\cdot \left(l+\left(\pi /2\right)\cdot \surd \left(a_{\max}/\pi \right)\right)\right)$$

Production of graphs and statistical and principle component analysis (PCA) were undertaken using R software (R Team, 2021).

To determine whether differences in rice SD impacted on recovery to drought, we utilised a generalised linear mixed‐effects model (GLMM) fit with a Poisson distribution. This was performed using the glmer function from the lme4 R package (Bates *et al*., 2015). The number of newly emerging leaves was set as the response variable, with SD groupings set as a fixed effect. Groupings were assigned as transgenics, low SD or medium to high SD based on SD values in Fig. 1. To account for variation between different varieties, variety was set as a random effect. To determine whether newly emerging leaf number was different between the three SD groupings we used a likelihood‐ratio test (LRT; ‘drop1’ function). The LRT compared the null GLMM, which had only random effects, with an identical model that also included SD grouping as a fixed effect. We targeted SD categories rather than individual varietal SD as we wanted test how categorical differences in SD impacted on drought responsiveness.

### Inductively coupled plasma‐mass spectrometry

Sample preparation was as described previously (Danku *et al*., 2013). Dried plant material was digested with 1 ml concentrated nitric acid (trace metal grade, Fisher Chemicals) spiked with Indium (internal standard) in dry block heaters (SCP Science; QMX Laboratories, Dunmow, UK) at 115°C for 4 h. Samples were then diluted to 10 ml with Milli‐Q Direct water (18.2 MΩ cm; Merck Millipore) and analysed using ICP‐MS (NexION 2000; PerkinElmer, Waltham, MA, USA) in the collision mode (He). Reference material (pooled samples) was run to correct for variation within ICP‐MS analysis run. Calibration standards were prepared from single element standards solutions (Inorganic Ventures; Essex Scientific Laboratory Supplies Ltd, Essex, UK). The final element concentrations were obtained by normalising concentrations to sample dry weight.

## Results

### Rice SS and density are negatively correlated and contribute to operating and calculated anatomical maximum stomatal conductance (*g*_smax_)

By overexpressing an EPF, we previously showed that reducing SD lowers rice water consumption and thus improves iWUE and drought avoidance (Caine *et al*., 2019; Mohammed *et al*., 2019). Here screened 72 traditionally bred rice varieties alongside two independently transformed *OsEPF1oe* lines to survey and compare stomatal traits between genetically engineered plants and a non‐transgenic population (Fig. S1; Table S1). Although SD differed widely across the collection, no traditionally bred varieties had a mean SD as low as IR‐64 *OsEPF1oe* transgenic rice lines. We did observe a negative correlation between SS and SD within the full collection of traditionally bred varieties (*r* ^2^ = 0.17, *P* < 0.0001, Fig. S1), with lower SD varieties typically having larger SS (with the exception of *OsEPF1oe* lines), and higher SD varieties typically having smaller SS. To study how these differences in stomatal traits might affect abiotic stress responses to increased drought, salinity and rising temperature and leaf VPD, nine varieties were selected spanning the range of SS and SD alongside *OsEPF1oe* plants. We conducted a series of experiments, beginning by measuring stomatal morphology, gas exchange and pore size on the leaves of tillering rice plants (Fig. 1).

Assessment of SD on leaf 8 of tillering rice (28–33 DPG) revealed traditionally bred varieties had mean SD values ranging from 233 to 393 stomata per mm^−2^, whereas the two independent *OsEPF1oe* lines had *c*. 180 stomata per mm^−2^ (Fig. 1a). As expected, a negative correlation between SS and SD was observed between varieties, with those with higher SD typically having smaller guard cells (*r* ^2^ = 0.38; *P* < 0.0001, Fig. 1b,c). *OsEPF1oe* lines had the lowest SD, with SS that was either equal to (*OsEPF1oeW*) or significantly larger (*OsEPFoe1S*) than IR‐64 control plants. This is the opposite to what we had previously found in IR‐64 plants at the leaf 5 stage where *OsEPF1oeS* SS was smaller than IR‐64 control plants (Caine *et al*., 2019). Despite having relatively large stomata, very low SD led to *OsEPF1oe* plants having the lowest calculated anatomical *g*_smax_ (Fig. 1d). Within the nine varieties selected, there were limited differences between *g*_smax_ values, but notably, the varieties with the two highest SD also had the highest mean *g*_smax_.

To investigate if operating *g*_sw_ followed a similar trend to *g*_smax_, we measured leaf *g*_sw_ using a porometer (Fig. 1e). While we found no overall correlation between operating *g*_sw_ and calculated *g*_smax_ across varieties (Fig. 1f), we did detect positive relationships between SD and operating *g*_sw_ (*r* ^2^ = 0.13; *P* < 0.01, Fig. 1g) and between SD and *g*_smax_ (*r* ^2^ = 0.24; *P* < 0.0001, Fig. S2a). We also identified a weak negative relationship between SS and *g*_sw_ (*r* ^2^ = 0.09; *P* < 0.02, Fig. 1h), but there was no significant correlation between SS and *g*_smax_ (*r* ^2^ = 0.04; *P* = 0.11, Fig. S2b). Despite very low calculated *g*_smax_, both *OsEPF1oe* lines were able to maintain a similar operating *g*_sw_ compared to some of the traditionally bred varieties, despite SD being *c*. 30% lower (Fig. 1a,d,e); While low SD was typically linked with low operating *g*_sw_, UCP‐188 bucked this trend having the equal highest *g*_sw_ (Fig. 1a,d). Our results suggested that factors other than SS and SD drove the differences in operating *g*_sw_, so we next assessed stomatal pore area. Overall, there was a robust correlation (*r* ^2^ = 0.47; *P* < 0.0001) between operating *g*_sw_ and the extent of stomatal opening across the nine selected varieties (Fig. 1i,j). Rates of stomatal water loss were explored by assessing whole‐plant surface temperatures as a proxy for evaporative transpiration – with lower temperatures indicating higher water loss. Thermal imaging confirmed first that the open‐pored (large SS) UCP‐188 and (high SD) C‐7306 varieties were cooler respectively than the Sathi and Bharathy varieties which had equivalent conformations of SS and SD. These similarities between surface temperatures and leaf *g*_sw_ values, indicate that in addition to SS and SD, that stomatal pore aperture (and perhaps other root and vascular features) can differ substantially between rice varieties and this also has the potential to greatly influence water loss (Fig. 1i–l).

**Fig. 2:**
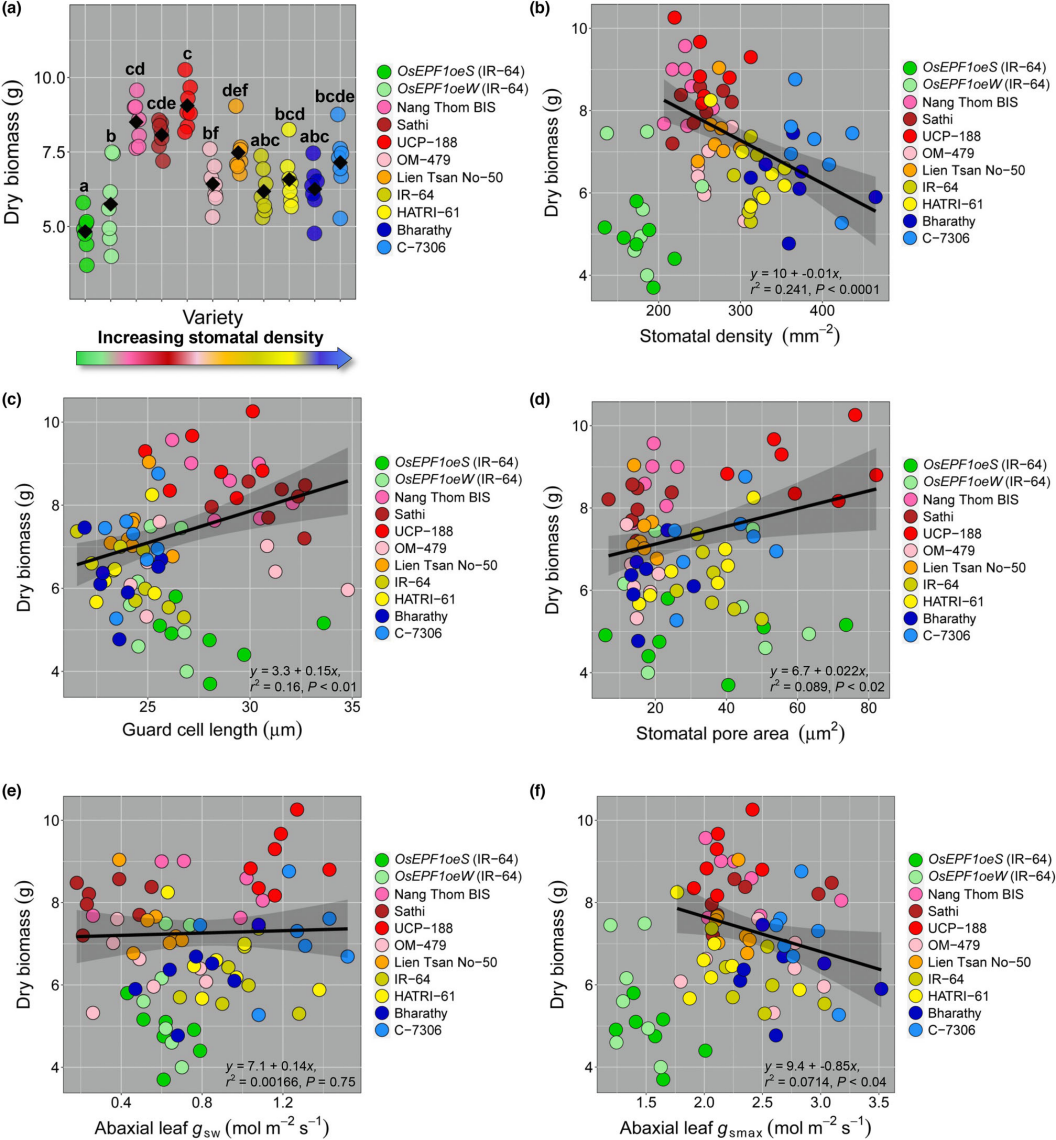
The relationships between rice (*Oryza sativa*) aboveground biomass and stomatal size (SS), stomatal density (SD) and leaf gas exchange parameters during vegetative tillering. (a) Dry plant biomass. (b–f) Regression analysis conducted between aboveground biomass and (b) SD, (c) SS (guard cell length), (d) stomatal pore area, (e) leaf stomatal conductance (*g*_sw_) and (f) calculated anatomical maximum stomatal conductance (*g*_smax_). Different letters indicate a significant difference between the means (one‐way ANOVA, Tukey HSD test, *P* < 0.05). Black diamonds represent means. *OsEPF1oe* plants are excluded from regression analyses. *n* = 7 plants.

To investigate if the differences in stomatal morphology and physiology (Fig. 1) were associated with overall plant growth, we measured vegetative‐stage aboveground biomass at 35 DPG (Fig. 2). Within the nine selected varieties, the low SD varieties typically had greater biomass than plants with higher SDs (Fig. 2a). The exceptions to this were the *OsEPF1oe* transgenic plants. With *OsEPF1oe* transgenic lines excluded from regression analyses, a moderate negative correlation was observed between SD and plant biomass (*r* ^2^ = 0.24; *P* < 0.001), and a weaker positive relationship was detected between SS and biomass (*r* ^2^ = 0.16*; P* < 0.01) (Fig. 2b,c). We also found that pore area was weakly associated with greater plant biomass (*r* ^2^ = 0.09; *P* < 0.02) (Fig. 2d), but no such relationship was detected for operating *g*_sw_ (Fig. 2e). Assessment of *g*_smax_ values revealed a very weak negative correlation with plant biomass (*r* ^2^ = 0.07; *P* < 0.04) (Fig. 2f), with smaller plants typically having marginally higher *g*_smax_ values, although this was not the case for *OsEPF1oe* plants.

To show potential associations between biomass, stomatal and gas exchange features of data presented (Figs 1, 2), a PCA was undertaken (Fig. S3). Principle component (PC) 1 is influenced mostly by SS and SD while PC2 is influenced by biomass, pore area and SD. Along PC1 and PC2, we observe a separation of the UCP‐188 variety (Fig. S3a,b). Along PC3, which is also influenced by biomass and pore area, a separation of both *OsEPF1oe* lines is observed. These findings re‐iterate that pore area and biomass are important features that contribute to the distinct performance differences we have observed in Figs 1 and 2.

### The association of stomatal traits on rice resilience to drought stress

**Fig. 3:**
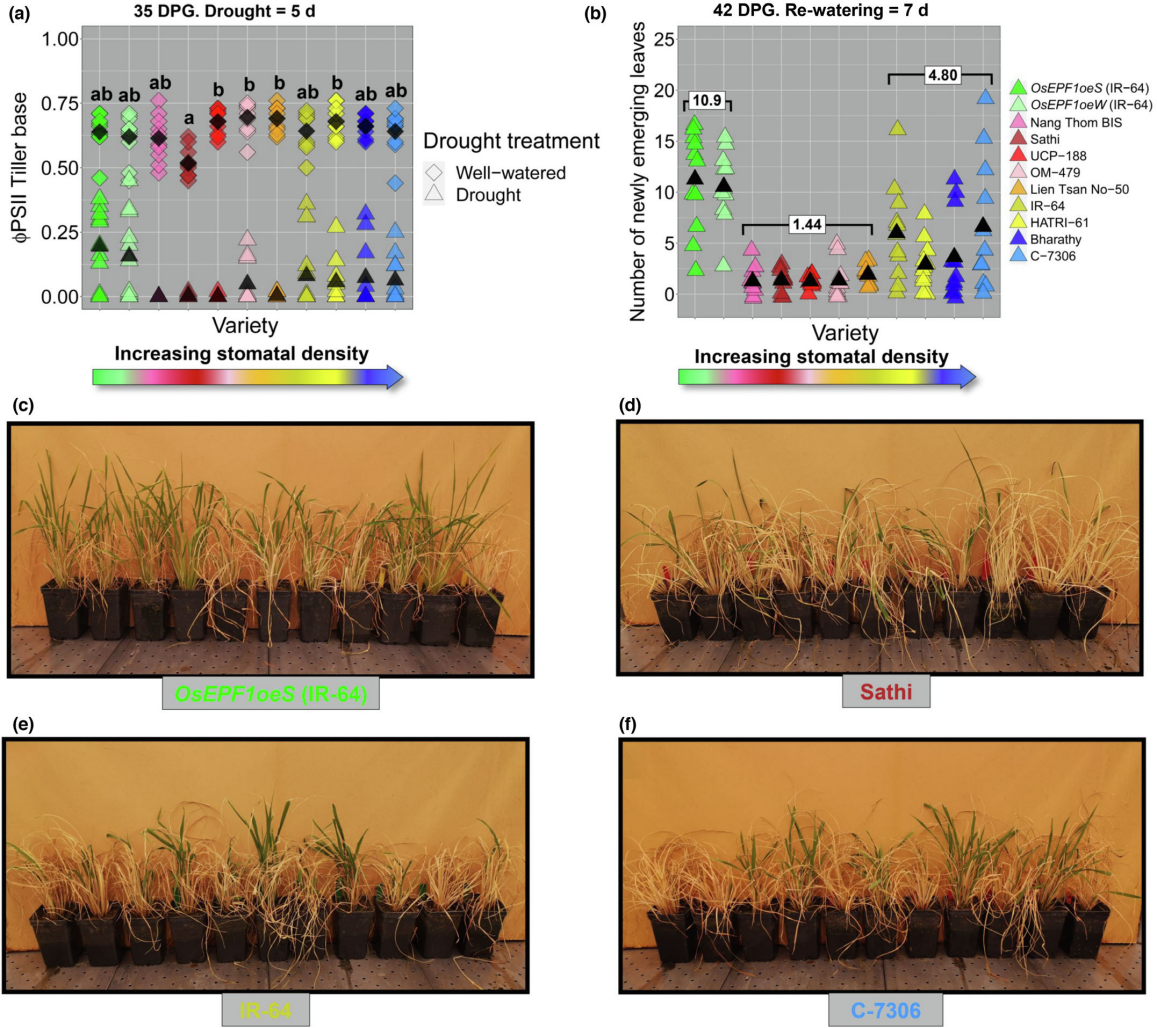
Natural rice varieties (*Oryza sativa*) with medium to high stomatal density (SD) and smaller stomatal size (SS) respond better to drought than lower SD large SS varieties. (a) Drought responses assessed at the tiller base using Φ PSII to measure plant health. (b) Number of new regenerative leaves 1 wk after re‐watering. (c–f) Examples of varieties with (c) very low SD (*OsEPF1oeS*), (d) low SD and large SS (Sathi), (e) medium SD and small SS (IR‐64), and (f) high SD and small SS (C‐7306) recovering from drought at 42 d, 1 wk after re‐watering. In (a), different letters indicate significant differences between the means of well‐watered samples (one‐way ANOVA, Tukey HSD test, *P* < 0.05). To assess drought responsiveness in (b), a generalised linear mixed model (GLMM) was used to compare the grouped SDs of transgenic (*OsEPF1oeS* and *OsEPF1oeW*), low SD (Nang Thom Bis, Sathi, UCP‐188 and OM‐479) and medium to high SD (IR‐64, HATRI‐61, Bharathy and C‐7306) varieties. Newly emerging leaves differed significantly between grouped varieties (GLMM, LRT; χ^2^ = 26.62, *P* < 0.00001). Black diamonds and triangles represent means for well‐watered and droughted varieties, respectively. SD category means are included in white boxes above the grouped varieties. *n* = 10–11 plants.

To understand how the nine selected varieties might compare to the *OsEPF1oe* plants when exposed to abiotic stress, we first imposed a 5‐d drought from 30 DPG (Fig. 3). Chlorophyll fluorescence measurements were taken to assess the efficiency of Photosystem II (ΦPSII) using a drop in the ΦPSII value to indicate plant stress (Caine *et al*., 2019).

Fig. 3 shows that varieties with higher SD and smaller SS respond better to drought than lower SD and large SS varieties. Under well‐watered conditions, the *OsEPF1oe* lines and most of the selected traditionally bred varieties shared similar ΦPSII values at the tiller base. Following 5 d of severe drought treatment, the ΦPSII values of all traditionally bred varieties and *OsEPF1oe* lines were dramatically lower than in well‐watered controls. Varieties with low SD (mostly with large stomata: Nang Thom BIS, Sathi, UCP‐188 and OM‐479) did not perform well, with most exhibiting ΦPSII values of 0 at the end of the drought (36 out of 44 plants), suggesting that the tillers of these plants were severely stressed (Fig. 3a). Plants with medium to high SDs (typically with smaller SS: IR‐64, HATRI‐61, Bharathy and C‐7306) performed better, with just over half of plants recording ΦPSII values of 0 (25 out of 44). Although there was variability between individual plants, the *OsEPF1oe* lines fared best and maintained the highest mean ΦPSII values (only 4 out of 22 plants displayed a ΦPSII value of 0).

All plants were then re‐watered and new leaf growth was assessed after 7 d (Fig. 3b–f). This revealed that the 35 DAG ΦPSII measurements were a good indicator of drought resilience. The number of newly emerging leaves differed significantly between transgenics, low SD and medium to high SD grouped varieties (GLMM, LRT; χ^2^ = 26.62, *P* < 0.00001; Fig. 3). Natural varieties with lower SD recovered the slowest and had the fewest new leaves (averaging 1.44 new leaves per plant). Medium and high SD varieties recovered quicker (averaging 4.8 new leaves) and transgenic *OsEPF1oe* plants displayed the fastest recovery (averaging 10.9 new leaves).

### OsEPF1oe lines have enhanced salt tolerance

To understand whether stomatal traits can influence how plants perform under saline conditions, we first compared the performance of the *OsEPF1oe* to IR‐64 control plants grown in 20 mM NaCl.

**Fig. 4:**
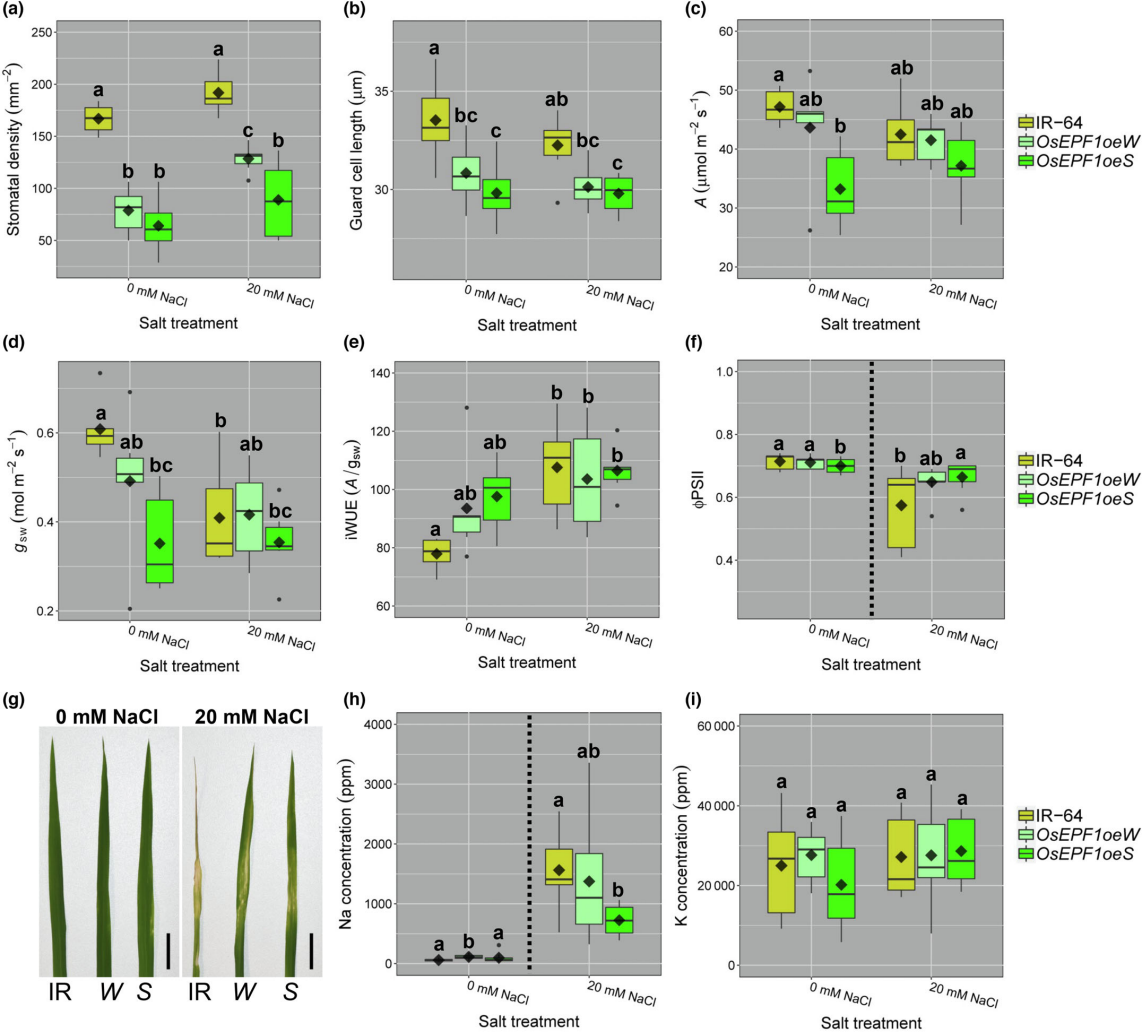
*OsEPF1oe* plants with reduced stomatal density (SD) display increased salinity tolerance during seedling and tillering stages. Abaxial leaf 5 (a) SD and (b) stomatal size (guard cell length) of fresh water and salt grown IR‐64 and *OsEPF1oe* plants at 19–21 d post germination (DPG). Fresh water and salt grown rice (*Oryza sativa*) plant gas exchange measurements of (c) carbon assimilation (*A*), (d) stomatal conductance (*g*_sw_) and (e) intrinsic water‐use efficiency (iWUE). (f) Φ PSII leaf measurements of apical leaves at 28 DPG with (g) representative leaf images from fresh water and salinity treated IR‐64 (IR), *OsEPF1oeW* (*W*) and *OsEPF1oeS* (*S*) plants (Bar, 2 cm). (h) Sodium (Na) and (i) Potassium (K) concentrations in auxiliary leaves of 35 DPG tillering plants. Whiskers indicate the ranges of the minimum and maximum values and different letters indicate a significant difference between the means (two‐way ANOVA, Tukey HSD test, *P* < 0.05). For (f) and (h), two separate Kruskal–Wallis one‐way ANOVAs were performed due to unequal variances (*P* < 0.05). Black diamonds represent means and black dots are outliers. *n* = 6–7 plants.

Growth under saline conditions resulted in an increase in mean SD (19–21 DPG), with salt‐treated *OsEPF1oeW* seedlings having significantly more stomata per mm^−2^ than equivalent fresh‐water grown plants when leaf 5 seedlings were assessed (Fig. 4a). SS was unaffected by salt treatment, although *OsEPF1oeS* plants had the smallest SS regardless of treatment (Fig. 4b). (This follows SS trends in seedling leaves found by Caine *et al*. (2019) rather than in leaf 8 tillering plants in Fig. 1b.) Gas exchange analysis showed that *OsEPF1oeS* (but not *OsEPF1oeW*) had significantly reduced *A* and *g*_sw_ relative to IR‐64 when grown in fresh water (Fig. 4c,d). Under saline conditions, IR‐64 *g*_sw_ was greatly reduced, and the *A* and *g*_sw_ rates were no longer significantly higher than either *OsEPF1oe* line (Fig. 4c,d). The *g*_sw_ of *OsEPF1oe* plants remained low between freshwater controls and salt‐treated equivalents. The reduced *A* and *g*_sw_ of salt‐treated IR‐64 plants resulted in an increased iWUE, whereas for *OsEPF1oe* plants, iWUE did not significantly increase under saline conditions (Fig. 4e).

At 28 DPG, the continuing impact of salt uptake was investigated using ΦPSII values as a proxy for plant health (Fig. 4f). Under normal freshwater conditions, the ΦPSII of *OsEPF1oeS* (but not *OsEPF1oeW*) leaves were significantly lower than IR‐64. Salinity treatment had a more severe impact on IR‐64 than the *OsEPF1oe* lines and salt‐treated *OsEPF1oeS* had higher ΦPSII values than IR‐64, with visibly healthier leaves (Fig. 4f,g). At 35 DPG, the concentration of accumulated salt in auxiliary leaves was measured. The plants with the lowest SD accumulated significantly less salt in their leaves, with *OsEPF1oeS* salt‐grown plants having *c*. 50% lower Na than IR‐64 equivalents (Fig. 4h). However, we did not detect any differences in K levels across genotypes or treatments (Fig. 4i).

**Fig. 5:**
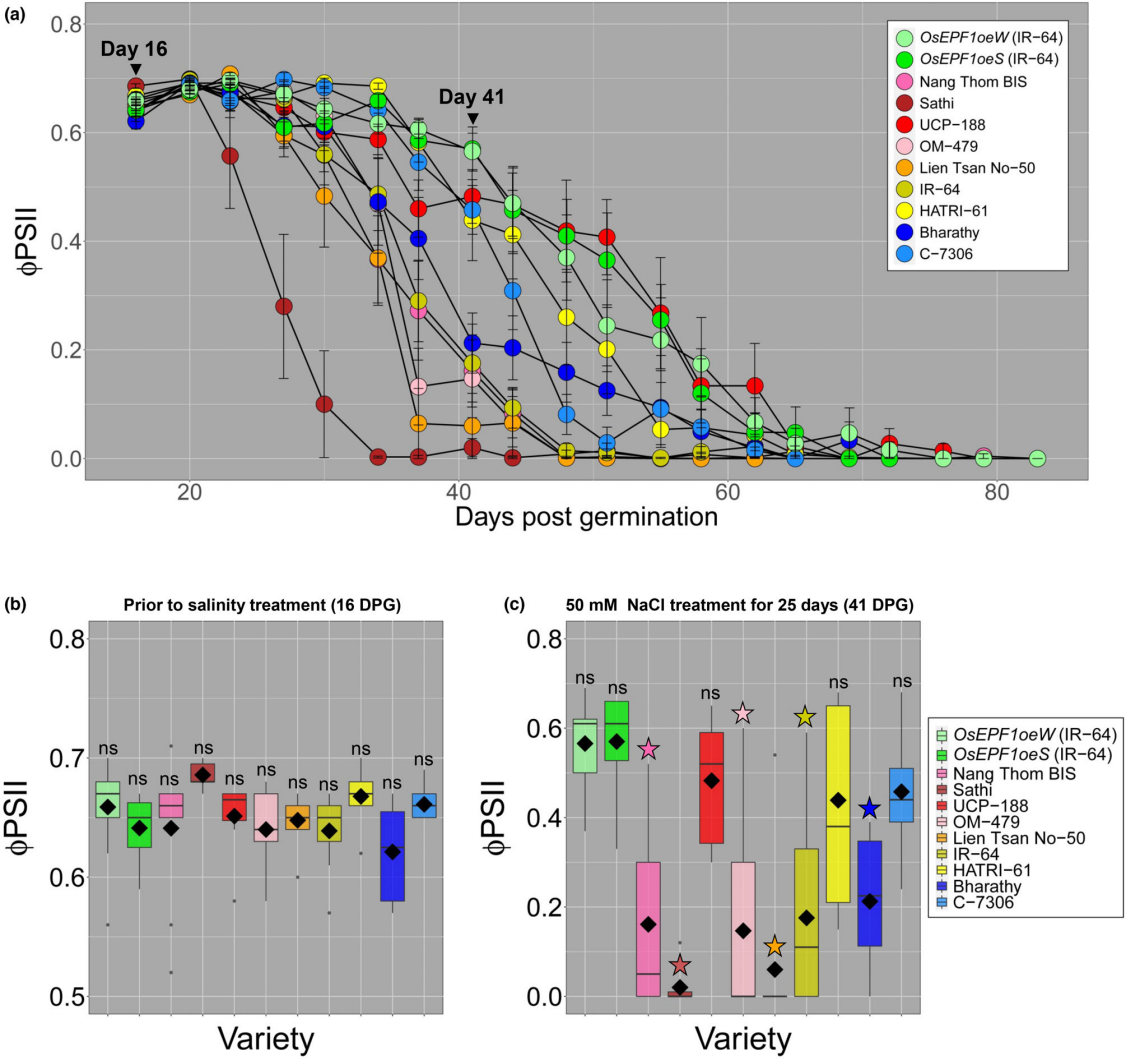
*OsEPF1oe* rice (*Oryza sativa*) maintains higher leaf ΦPSII values for equal or longer than all traditionally bred rice varieties. (a) Apical leaf ΦPSII measurements of the nine natural varieties and two *OsEPF1oe* lines grown in 50 mM NaCl solution for 67 d. Vertical lines show one SE (b) ΦPSII values before salt treatment at day 16 (ns, not significant). (c) ΦPSII values on the apical leaf at 41 d post germination, 25 d after the commencement of the salt treatment. For (b, c), whiskers indicate the minimum and maximum values and stars indicate a significant difference from salt tolerant *OsEPF1oeW* (one‐way ANOVA, Tukey HSD test, *P* < 0.05). Black diamonds represent means. *n* = 7–9 plants.

Next, we investigated how the nine selected traditionally bred varieties and two transgenic lines performed under exposure to 50 mM NaCl from 16 DPG onward. We measured ΦPSII every 3–4 d for the next 67 d (Fig. 5).

Fig. 5 demonstrates that before the start of salt treatment (16 DPG), there was no significant differences between *OsEPF1oeW* leaf ΦPSII values and any of the other varieties assessed (Fig. 5a,b). After 25 d of salt treatment (41 DPG), six out of nine varieties (including IR‐64) showed significant reductions in ΦPSII (Fig. 5a,c). Post 41 DPG, *OsEPF1oe* plant leaves continued to show a slower decline in health than the majority of other varieties, with the exceptions being UCP‐188. Within the selected varieties, neither SS nor SD appeared to be associated with the degree of salinity tolerance.

### Stomatal responses to raising temperature and associated leaf VPD

**Fig. 6:**
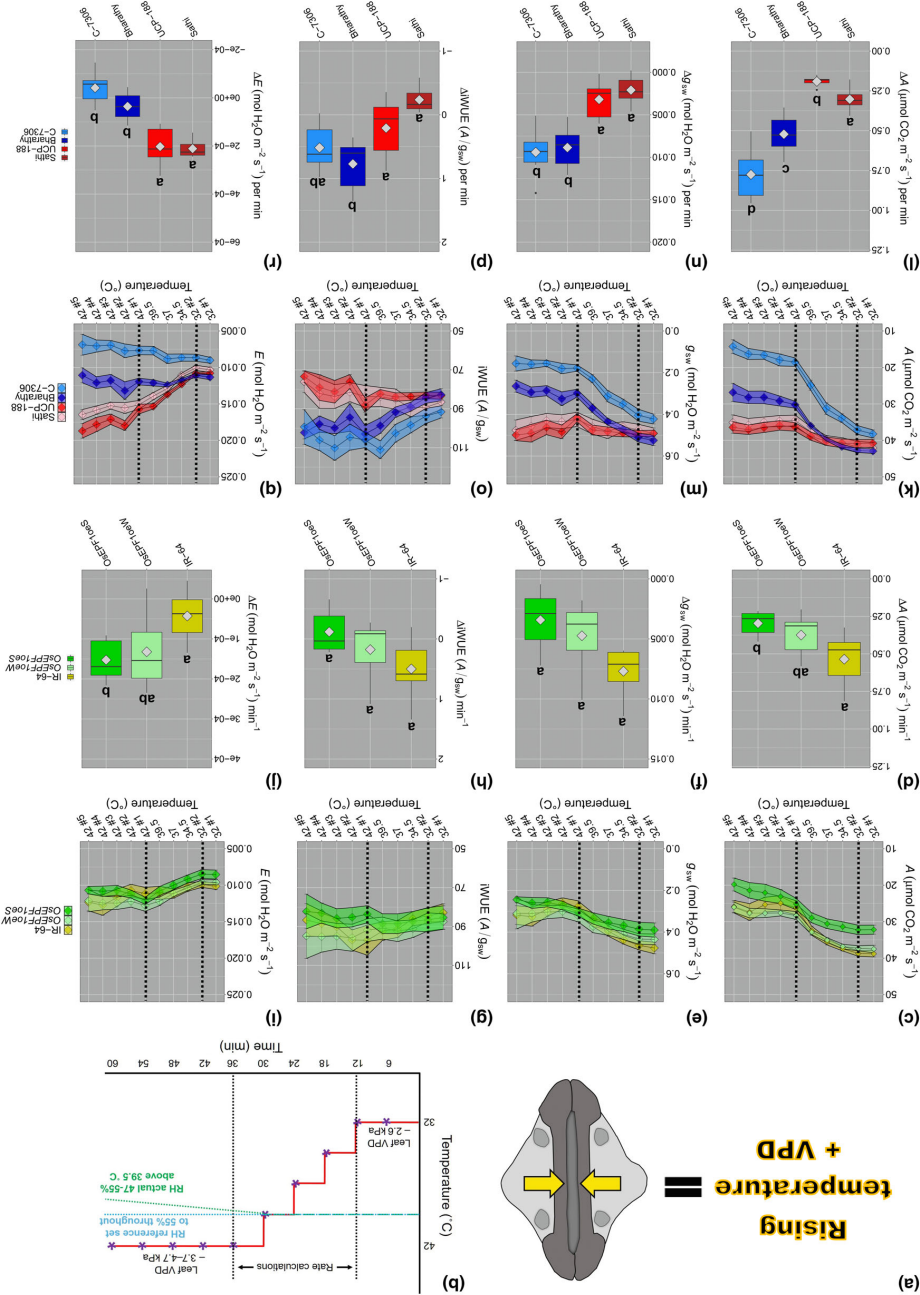
Dynamic stomatal responses to rising temperature lead to large alterations in rice (*Oryza sativa*) photosynthesis and water‐use efficiency. (a) Increasing temperature coupled with increasing leaf vapour pressure deficit (VPD) often causes stomatal closure. (b) Program used in Li‐Cor 6800 gas analysers used to study stomatal responses to temperature and leaf VPD. At *c*. 39.5°C and more, it was not possible to maintain relative humidity (RH) at 55% owing to additive RH being unable to reach the set point for a number of the different varieties surveyed (see also Supporting Information Fig. S4). Purple stars indicate recorded data points. Parallel vertical dotted lines indicate period used to calculate rate changes. (c–j) Comparison of IR‐64 control plants and *OsEPF1oeW/OsEPF1oeS* transgenics investigating leaf 5 responses to raising temperature and VPD. (c, d) Carbon assimilation (*A*) responses over 1‐h duration and (d) rate of change per minute during the 24‐min incline period. (e, f) Equivalent stomatal conductance (*g*_sw_) responses and rates of change over 24‐min period. (g, h) Corresponding intrinsic water‐use efficiency (iWUE) responses and rates of change and (i, j) transpiration (*E*) responses and rates of change. (k–r) Comparisons between low SD, large SS varieties Sathi and UCP‐188 and high SD, small SS varieties Bharathy and C‐7306. (k) *A* responses over 1‐h assay and (l) rate of change per minute during the 24‐min incline period. (m, n) Equivalent *g*_sw_ responses and rates of change. (o, p) Corresponding iWUE responses and rates of change and (q, r) *E* responses and rates of change. Ribbons highlight SE of the mean. Boxplot whiskers indicate the minimum and maximum values and different letters indicate a significant difference between the means (one‐way ANOVA, Tukey HSD test, *P* < 0.05). Grey diamonds represent means. *n* = 7–8 plants.

At higher temperatures, stomatal open to increase plant cooling. Paradoxically however, as temperatures rise, the amount of water the air can hold also increases and this often leads to rising VPD causing stomatal closure (Grossiord *et al*., 2020). We investigated how increases in temperature (and associated leaf VPD) impacted stomatal dynamics by undertaking gas exchange experiments that either rapidly increased temperature (Figs 6, S4) or maintained at a constantly high temperature (Fig. S4). These experiments were performed at constant (or slightly reduced) RH that meant that leaf VPD increased as temperature rose. For the rapid temperature increase experiments, assays were conducted over a 1‐h duration, with a 10°C increase inside the chamber (from 32 to 42°C), between 12 and 36 min into the experiment. Over that 24‐min period, leaf VPD increased from *c*. 2.6 to 3.7–4.7 kPa (Figs 6b, S4). Variation in leaf VPD and chamber RH at higher temperatures (≥ 39.5°C) were predominantly caused by reductions in water flow caused by increased stomatal closure and by an inability of the infrared gas analyser (IRGA) to maintain a constant RH (Fig. S4). At the leaf 5 stage, we found that *OsEPF1oeS* plants, but not *OsEPF1oeW* plants, had significantly smaller SS than IR‐64 controls when grown under high light (1500 μmol m^−2^ s^−1^ PAR) (Fig. S4).

We first assayed IR‐64 and *OsEPF1oe* plants and found that all plants showed a significant decrease in *A* as temperature and leaf VPD increased to 42°C (ANOVA, *P* < 0.0001, Fig. 6c), but the rate reduction in *A* occurred significantly more slowly for *OsEPF1oeS* than in IR‐64, but not *OsEPF1oe*W (Fig. 6d). Like *A*, *g*_sw_ also reduced with increasing temperature and leaf VPD (ANOVA, *P* < 0.001, Fig. 6e); however, there were no significant differences in rate changes between genotypes, despite a clear trend for a slower rate in *OsEPF1oeS* (Kruskal–Wallis one‐way ANOVA, overall *P* = 0.056) (Fig. 6f). The slower decreases in *A* and (to some extent *g*_sw_) resulted in a trend towards lower iWUE for *OsEPF1oeS* during the temperature incline relative to IR‐64 (Fig. 6g,h) (Kruskal–Wallis one‐way ANOVA, overall *P* = 0.1052). Conversely, *E* responses generally increased across all genotypes with *OsEPF1oeS* plants having increased rates of *E* relative to IR‐64 during the temperature incline, peaking at the end of the increase. The *E* of IR‐64 peaked later and by the end of the 1‐hour experiment reached a similar level to *OsEPF1oe* plants (Fig. 6i,j).

We next compared the stomatal responses of two selected varieties which had lower SD and larger SS (Sathi and UCP‐188), with two varieties which had higher SD and smaller SS (Bharathy and C‐7306) (Figs 6k–r, S4). There were no significant differences in *A* or *g*_sw_ at 32°C between the two low SD, large SS varieties and the two high SD, small SS varieties at the beginning of the experiments, but large differences were detectable in response to rising temperature and leaf VPD (Fig. 6k–n). Specifically, high SD with small SS varieties showed significantly faster rates of reduction comparatively to low SD and large SS varieties for both *A* and *g*_sw_ as temperature and leaf VPD rose (Figs 6k–n, S4). This faster high SD, small SS response resulted in large increases in iWUE (Fig. 6g,o–p). We also detected striking differences in *E*, with varieties that had low SD and large SS displaying a rapid increase in *E* as temperature and leaf VPD increased, which was in contrast to plants with high SD and small SS that showed little change (or a slight drop) in *E* (Fig. 6q,r). Thus, the two high SD, small SS varieties reduced *A* and *g*_sw_ relatively quickly in response to rising temperature and leaf VPD, whereas for low SD varieties with large SS, *A* and *g*_sw_ remained higher and this led to *E* increasing.

The experiments in Fig. 6 highlight that high SD, small SS rice varieties were unable to maintain high *A*, *g*_sw_ and *E* when exposed to increasingly high temperature and rising leaf VPD. It is possible that this effect was partly due to a transient drop in chamber RH during the rapid closure of small stomata that contributed to leaves having higher VPD (green dotted line Fig. 6b, see also Fig. S4). We conducted a subsequent experiment where plants were held under steady‐state conditions at 39°C, with chamber RH maintained at 55%, and this led to more similar leaf VPD values between varieties (Fig. S4). These conditions captured the maximum point where all four different rice varieties were able to maintain steady‐state conditions. This removed any additional stress on plant leaves caused by insufficient supply of RH (as observed in our Fig. 6). Confirming the dynamic response experiments (Fig. 6), plants with low SD and large SS maintained a higher *A* and *g*_sw_ than those with high SD and small SS. Reductions in *A* and *g*_sw_ again had the opposite effect on iWUE, with high SD, small SS plants typically having higher iWUE at the expense of *A* and *g*_sw_ (Fig. S4). These changes also appeared to be entwined with an increased capability of plants with low SD and large SS to increase *E*, whereas for plants with high SD and small SS, *E* stayed low (Fig. S4). These combined responses led to plants with lower SD and larger SS having lower leaf temperatures based on calculated energy balance (Fig. S4).

## Discussion

### SS and density impact on plant gas exchange

A low SD (often associated with larger SS) and/or small SS (often associated with high SD) have frequently been correlated with improvements in water‐use and/or drought avoidance (Drake *et al*., 2013; Hepworth *et al*., 2015; McAusland *et al*., 2016; Dittberner *et al*., 2018; Kardiman & Ræbild, 2018; Caine *et al*., 2019; Durand *et al*., 2019; Mohammed *et al*., 2019). In this study, we took a stomatal‐focused approach to investigate how differences in SS and SD can affect rice performance under several separate abiotic stresses. Anatomical screening of stomatal properties across 72 rice varieties and 2 transgenic lines identified significant variation in both SS and SD (Fig. S1). *OsEPF1oe* lines had the lowest SD, suggesting that finding traditionally bred rice varieties with SDs and *g*_smax_ values equivalent to *OsEPF1oe* is unlikely.

Previous research has shown that high SD (often accompanied by small SS) can lead to a higher calculated *g*_smax_ (Franks & Beerling, 2009), and this is associated with higher operating *g*_sw_ and greater responsivity of stomatal apertures to environmental changes (Franks *et al*., 2012; Drake *et al*., 2013; Bertolino *et al*., 2019). Our results only partially support these findings. We observed the expected correlations between SD and both *g*_sw_ and *g*_smax_, with the two varieties with highest SD and small SS (Bharathy and C‐7306) also having the highest average *g*_smax_ values (Fig. 1). The lack of correlation between operating *g*_sw_ and *g*_smax_ under well‐watered conditions suggests that factors other than SD and SS might also be impacting on the operating *g*_sw_ we observed. Our results could have been impacted by growth chamber light intensity (1500 μmol m^−2^ s^−1^ PAR), which while high, was not saturating (*c*. 2000–2200 μmol m^−2^ s^−1^ PAR) (Murchie *et al*., 2022), and so not all stomata may have been as open possible. Nevertheless, we identified one naturally bred variety, UCP‐188, that behaved differently to lines with similar SS and SD, and this clearly impacted on our *g*_sw_–*g*_smax_ regression analysis (see Fig. 1e,f). Despite a low *g*_smax_ (driven by low SD), UCP‐188 had the equal highest operating *g*_sw_ caused by stomatal pores that were wide open (Fig. 1j). It is unclear what physiological mechanisms were driving stomatal opening in the UCP‐188 variety and this represents an interesting area of future study.

The correlations we observed between plant biomass and SD, and plant biomass and SS suggest that stomatal characteristics at the tillering stage are reasonable predictors of plant size (Fig. 2). Plants with low SD and large SS typically had higher biomasses (with the exception of *OsEPF1oe*), and plants with a high SD and small SS typically had lower biomasses. Importantly, differences in biomass have the potential to exacerbate differences in water‐use, as plants with a larger surface area often require more water (Feldman *et al*., 2018) and may also close their stomata more slowly than smaller plants (Drake *et al*., 2013; McAusland *et al*., 2016; Lawson & Vialet‐Chabrand, 2019). Evidence does suggest, however, that plants with larger stomata have higher operating WUE (Drake *et al*., 2013), which could potentially mitigate a larger plant size effect.

### Stomatal associations with drought tolerance

Our drought stress experiments showed that traditionally bred, smaller biomass varieties with high or medium SD and small SS, maintained higher ΦPSII values for longer during drought, and also recovered faster post‐drought compared with traditionally bred varieties with low SD and large SS. In poplar, Durand *et al*. (2019) show that high SD and small SS increases stomatal closure responses under well‐watered conditions and also often during sustained drought when plants are grown under glasshouse conditions. However, high SD, small SS in poplar was associated with higher plant biomass than low SD, large SS plants grown under well‐watered conditions, which is opposite to our well‐watered rice biomass results. During a sustained drought, high SD, small SS poplar had reduced biomass, being only equal to droughted low SD, large SS poplar plants (Durand *et al*., 2019). This implies that high SD, large SS did not aid in improved drought resilience in Poplar in respect to biomass whereas we did detect a better drought response in rice in respect to regeneration. Some of the disparity between findings could have been caused by differences in the type of drought administered (rapid drought in rice vs sustained drought in poplar), but together both sets of findings highlight how larger plant biomass is not necessarily favourable to improved drought resilience.

Both *OsEPF1oe* lines with very low SD plants had lower biomasses, and this combinations of traits resulted in *OsEPF1oe* plants maintaining the highest ΦPSII at the end of the drought. This was despite *OsEPF1oe* plants displaying similar operating *g*_sw_ levels as other low SD, large SS varieties under well‐watered conditions (Fig. 1e). Together, our results suggest that the ideal combination of traits for growth of rice under drought conditions would be small plant size together with either a very low SD (leading to a very low *g*_smax_) or a higher SD combined with a small SS (leading to a high *g*_smax_ and greater stomatal, responsivity).

### Stomatal contributions to salinity tolerance

Modelling of root water and solute uptake has suggested that passive transport and uptake of water and solutes into plants may be negligible (Foster & Miklavcic, 2017). However, recent research in rice suggests that reduced SD and *g*_sw_, caused by increased activity of a histone deacetylase, improved both drought and salinity tolerance (Zhao *et al*., 2021). In our study, we tested this potential relationship between SD and salt tolerance and found that *OsEPF1oe* plants with reduced SD showed improved performance with increasing salinity (Fig. 4). *OsEPF1oe* plants maintained higher leaf ΦPSII values when grown in salt water, and also accumulated less than half the amount of Na^+^ in leaves after approximately 5 wk of growth (Fig. 4). Salt toxicity often leads to deficiencies in other elements such as K^+^ (Wang *et al*., 2013) but we did not detect this in *OsEPF1oe* plants or controls. We compared the performance of traditionally bred varieties against *OsEPF1oe* and found that, as with drought experiments, *OsEPF1oe* plants performed well, maintaining leaf ΦPSII for the equal longest time of all varieties surveyed. By contrast, other varieties with low SD and large SS performed the least well (with the exception of UCP‐188) (Fig. 5a,c).

### Stomatal responsiveness to rising temperature and associated leaf VPD

Rising temperatures combined with increasing VPD has the potential to shut stomata at a time when plants might otherwise utilise transpiration‐driven evaporative cooling to maintain a high photosynthetic output (Urban *et al*., 2017; Yuan *et al*., 2019; Grossiord *et al*., 2020). Comparisons between IR‐64 and transgenic plants revealed that *OsEPF1oeS* leaf 5 with very low SD and small SS had slower rates of *A* decline at higher temperature as leaf VPD increased, and this was linked with a trend towards a decreased rate of *g*_sw_ change per minute (Fig. 6). These slower reductions in *A* and operating *g*_sw_ were coupled with increasing rates of *E*, suggesting that *OsEPF1oeS* leaves with very low SD lost more water in comparison to IR‐64 as temperature (and leaf VPD) increased. This suggests that having a combination of vastly reduced SD, and small SS may be detrimental when temperature and leaf VPD are constantly fluctuating. When guard cell turgor pressure increases lead to larger stomatal apertures, this can produce *g*_sw_ values that are above a plant operating dynamic range, and this can lead to reduced stomatal responsivity (Dow & Bergmann, 2014). Our research here and previously (Caine *et al*., 2019) has shown that *OsEPF1oeS* plants open stomatal apertures wider to compensate for reduced SD, and so this could have contributed to the slower responsivity of *OsEPF1oeS* plants we observed during our experiments.

Small SS is often associated with faster stomatal responsiveness, which in turn can result in higher water‐use efficiency under fluctuating conditions (McAusland *et al*., 2016; Lawson & Vialet‐Chabrand, 2019; Durand *et al*., 2019; Inoue *et al*., 2021). However, if plants with small SS (often with high SD) were to close stomata rapidly on rising VPD, this could lead to detrimental reductions in *A* and evaporative cooling. Comparisons of leaf 5 responses of plants with opposing stomatal traits found that, unlike *OsEPF1oe* lines, that natural varieties with small SS (and higher SD) reduced their *g*_sw_ much more rapidly than plants with large SS and low SD (Fig. 6). These results confirm VPD literature in poplar under glasshouse conditions (Durand *et al*., 2019), but further research in poplar also showed that stomatal responses are environment dependent, as under field conditions plants with high SD and small SS no longer exhibited faster responses to VPD or irradiance (Durand *et al*., 2020). Accordingly, further temperature and VPD research conducted on rice in the field is an important next step to continue to investigate rice stomatal dynamics.

Small SS (and high SD) rice closed their stomata extremely efficiently when exposed to higher temperatures and rising leaf VPD. This reduced *g*_sw_ so effectively that IRGA equipment was unable to maintain RH levels within the leaf chamber, whereas for plants with larger SS and low SD, RH was maintained (Fig. S4g). This rapid stomatal closure of small SS, high SD plants improved iWUE as temperature and leaf VPD increased, but this was at the expense of *A*. By contrast, varieties with larger SS and low SD maintained higher rates of *A* and *g*_sw_, with *E* increasing markedly as a consequence. These differing stomatal behaviours could well help contribute to enabling different rice varieties to inhabit and thrive under different environments. For example, the fast responses of small SS (potentially alongside high SD) could be beneficial in water restricted conditions, but higher levels of *E* associated with plants with larger SS (and potentially lower SD) might be more beneficial under warm conditions if water is more freely available. This last point may be especially important considering that seed yield has been shown to often decrease by 10% or more for every 1 degree increase in temperature above seasonal averages (Lyman *et al*., 2013).

### Effects on stomatal development

Our results support previous observations (Zhang *et al*., 2019) showing that rice SD and SS are, in general, negatively correlated (Figs 1, S1). During leaf development, epidermal cell division is usually synchronised with cell expansion to achieve a specific SS and SD on a given leaf. This relationship is perturbed in *OsEPF1oe* seedlings, where plants with extremely low SD, typically have smaller SS at the leaf 5, seedling stage (Fig. S4) (Caine *et al*., 2019). However, by leaf 8, when plants are rapidly tillering, this is no longer the case, and *OsEPF1oe* plants had larger SS (than IR‐64) in their more mature leaves (Fig. 1). SD is also under developmental control, and in our experiments the leaves of mature plants had considerably more stomata than seedling leaves. Thus, the potency or nature of factors driving SS and/or SD regulation must change as a plant develops. Recently, a genome‐wide association study has identified genetic traits associated with both SS and SD that could be useful in breeding for the most appropriate SS and SD for a given environment in the future (Chen *et al*., 2020).

### Conclusion

Previously, we have shown that *OsEPF1oe* plants with extremely low SD have reduced water consumption with increased drought survivability, even at high temperatures. Here, we show that *OsEPF1oe* plants are also less susceptible to salinity toxicity, most probably because they accumulate salt at much lower levels. By screening a range of traditionally bred rice varieties, we show that varieties with low SD and large SS typically have lower operating *g*_sw_ when sufficient water is available, but this is often associated with increased biomass that could promote increased drought susceptibility. *OsEPF1oe* plants were physically smaller and yet had the lowest SD and *g*_smax_ of all varieties studied, traits which together appeared to positively impact on drought avoidance and increased salinity tolerance. Based on our screen, it seems unlikely that similar conformations of stomata with correspondingly small plant size will be found in traditionally bred rice varieties. This means that stomatal‐based stress resilience may best be obtained via genetic manipulation of stomatal development rather than via conventional breeding practices.

Assessment of temperature and leaf VPD responses suggest that strong overexpression of *EPF1* diminishes stomatal closure relative to IR‐64, leading to reduced short‐term iWUE. This slower stomatal responsiveness results in increased *E*. Conversely, traditionally bred plants with high SD and small SS greatly reduce *g*_sw_ as temperature and leaf VPD rise, preventing *E* increasing, and this may be beneficial for short‐term iWUE but detrimental to long‐term plant productivity and cooling. While *OsEPF1oe* plants showed enhanced resilience to multiple abiotic stresses, taken together our results highlight that there is unlikely to be an ideal SS and SD for all future climatic conditions, and thought must be given as to which conformation(s) of stomata will best suit a given growth environment.

## Competing interests

None declared.

## Author contributions

RSC, LTN and JEG designed the study. RSC, ELH, JS, PMF and SF undertook the experiments. RSC, JS, SF and MSK conducted the data analysis. LTN, PTN, HC and JEG contributed materials and advice. RSC, HC and JEG wrote the paper with comments from ELH, JS, PMF, SF, MSK, LTN and PTN. All authors read, commented on and approved the final version of the manuscript.

## Supporting information


**Fig. S1** Rice leaf 5 stomatal size and density screen of 72 traditionally bred rice varieties and 2 transgenic varieties.
**Fig. S2** Stomatal size and density relationships with stomatal conductance.
**Fig. S3** Principle component analysis (PCA) of stomatal, biomass and gas exchange data.
**Fig. S4** Rapid and steady‐state VPD responses of rice with differing stomatal size and density.
**Table S1** List of 72 rice varieties and corresponding stomatal size and densities.
Please note: Wiley is not responsible for the content or functionality of any Supporting Information supplied by the authors. Any queries (other than missing material) should be directed to the *New Phytologist* Central Office.

## Data Availability

Data available on request from the authors.
