# Supplementary material for: The influences of stomatal size and density on rice abiotic stress resilience
Source: New Phytol. 2023 Jan 11;237(6):2180–95. doi: 10.1111/nph.18704 (PMC10952745; doi:10.1111/nph.18704)
Supplement: Supplementary file 1 — Fig. S1 Rice leaf 5 stomatal size and density screen of 72 traditionally bred rice varieties and 2 transgenic varieties. Fig. S2 Stomatal size and density relationships with stomatal conductance. Fig. S3 Principle component analysis (PCA) of stomatal, biomass and gas exchange data. Fig. S4 Rapid and steady‐state VPD responses of rice with differing stomatal size and density. Table S1 List of 72 rice varieties and corresponding stomatal size and densities. Please note: Wiley is not responsible for the content or functionality of any Supporting Information supplied by the authors. Any queries (other than missing material) should be directed to the New Phytologist Central Office. [file NPH-237-2180-s001.pdf]

New Phytologist Supporting Information

Article title: The Influences of Stomatal Size and Density on Rice Abiotic Stress Resilience

Authors: Robert S. Caine, Emily L. Harrison, Jen Sloan, Paulina M. Flis, Sina Fischer, Muhammad S. Khan, Phuoc Nguyen Trong, Lang Nguyen Thi, Julie E. Gray, Holly Croft

Article acceptance date: 05/12/2022

Table S1. Stomatal size and density measurmentes of 72 varieties and 2 transgenic rice (*Oryza sativa* L.) lines assessed during screen**.** *n = 1-7*

Variety	Stomatal density (mm^{-2})	Guard cell length (µm)	Replicate number	Variety	Stomatal density (mm^{-2})	Guard cell length (µm)	Replicate number
Bharathy	232.0	29.22	4	OM-5629	149.8	27.87	6
Tep Hanh	212.4	27.13	1	Doc Phung Lun	148.4	29.02	5
Nang Du	197.7	30.07	2	HATRI-20	148.1	31.71	6
C-7306	196.1	26.68	2	HATRI-192	148.0	32.77	7
Cadung Gocong	185.0	27.30	5	Kharai Ganga	147.1	32.03	1
IARI-5823	178.9	28.29	4	Mot Bui Do	147.1	30.80	1
Lien Tsan No-50	177.8	25.31	5	OM-442	146.0	26.31	3
Gogo Putih	176.9	31.06	7	Jao Hom Nin	145.2	33.52	7
Nang Nhen Thom	174.6	28.94	7	CTG-1516	143.8	29.52	3
DNJ-155	174.0	31.12	4	OM-447	142.7	33.96	3
San Tan Thou	173.2	30.04	6	Nang Thom	142.2	34.71	2
DNJ-11	170.6	32.78	5	Nang Rum Trang	140.5	32.32	1
ARC-10362	170.6	29.39	5	OM-457	140.5	29.63	1
Hea Doh-4	169.9	29.29	4	OM-453	140.5	29.45	1
OM-6976	169.9	30.13	2	HATRI-35	140.5	27.44	6
HATRI-31	168.8	31.98	3	OM-472	139.4	29.74	3
Nang Quot	166.7	30.06	5	OM-456	138.9	29.77	2
Dinlaga	166.7	30.89	1	Suhasini	138.9	27.25	2
OM-9921	166.7	29.72	1	OM-4900	138.6	27.17	5
Langmanbi	166.7	32.62	2	Jhona-349	138.3	34.00	6
Sarjoo-50	165.6	30.79	6	Kalubala Vee	137.3	32.33	4
Khao Pahk Maw	164.1	34.32	5	Kakuya	137.3	29.20	1
Perrum Karruppan	164.1	30.38	5	Shiratama	136.4	32.62	4
Malagpit Pirurutong	161.8	32.22	6	3263	135.6	30.79	2
Doc Phung	161.2	31.23	3	Nang Bang Bentre	134.0	34.44	1
OM-455	161.2	30.99	3	DV-86	134.0	34.28	1
OM-476	160.1	29.85	1	OM-471	134.0	34.15	3
HATRI-608	160.1	30.74	2	Tunsart Thai-Lan	131.5	29.20	4
HATRI-50	158.8	30.05	5	UCP-188	127.5	35.13	3
DHARIA	158.0	29.88	6	Nipponbare	123.7	33.06	7
HATRI-603	155.8	32.62	3	LM1 Tanh Binh	122.5	31.03	6
Yeh Hua Chan	155.2	28.99	4	OM-446	120.9	31.94	1
Pilang Baybay	153.6	29.43	1	Sathi	119.3	36.10	6
OM-2517	151.3	29.44	7	OM-479	116.0	32.24	2
Nang Thom Bis	151.1	29.56	4	OM-11735	114.4	31.12	1
HATRI-61	150.3	29.36	6	OsEPF1oeW (IR-64)	71.4	27.48	7
IR-64	150.3	29.20	6	OsEPF1oeS (IR-64)	63.0	28.89	7

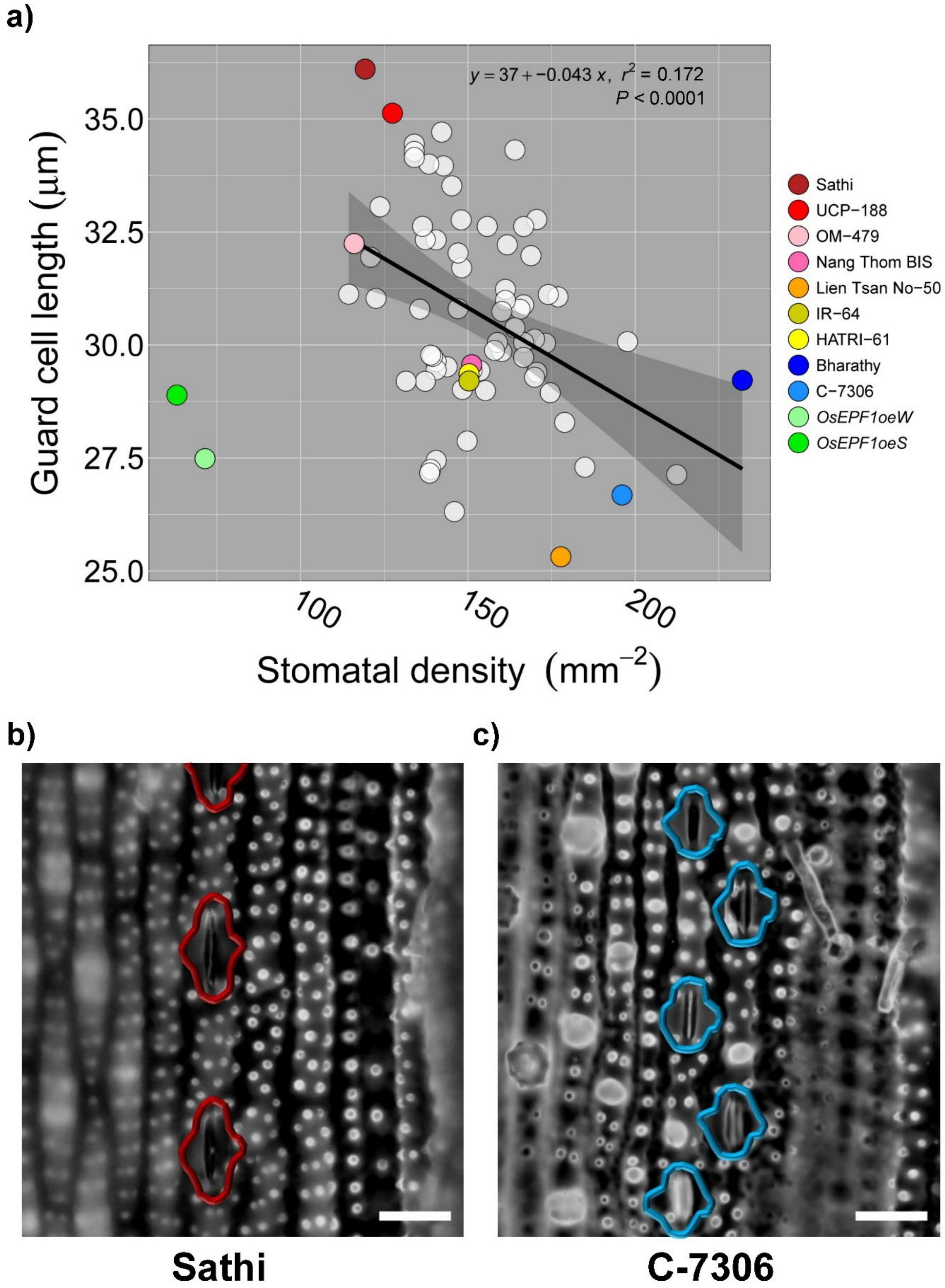

Fig. S1. Screening for stomatal size (SS) and density (SD) variation amongst 72 traditionally-bred rice (*Oryza sativa* L.) varieties and 2 transgenic *OsEPF1oe* lines on leaf 5. (**a**) SS and SD plot of assessed varieties and transgenics. The subsequently studied nine naturally-bred

varieties and two transgenic lines are highlighted by different coloured circles. White circles denote other screened varieties. See also Table. S1 for a full list of varieties and measurements. Representative epidermal images of SS and SD differences (**b**) low SD, large SS Sathi and (**c**) high SD, small SS C-7306 (bar, 25 μm).

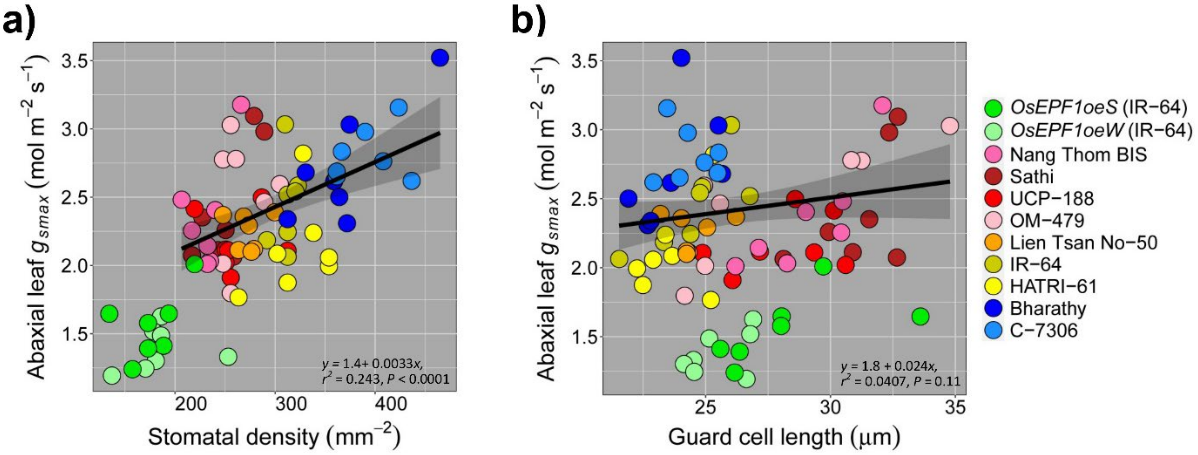

Fig. S2. Gas exchange relationships linked to Abaxial leaf stomatal size (SS) and density (SD). (**a**) Regression analyses conducted between (a) Calculated maximum anatomical stomatal conductance (g_{smax}) and SD and (**b**) abaxial g_{smax} and SS. Regression analysis and trend lines are based on linear models. *OsEPF1oe* plants are excluded from regression analyses. a-b, n = 7 plants.

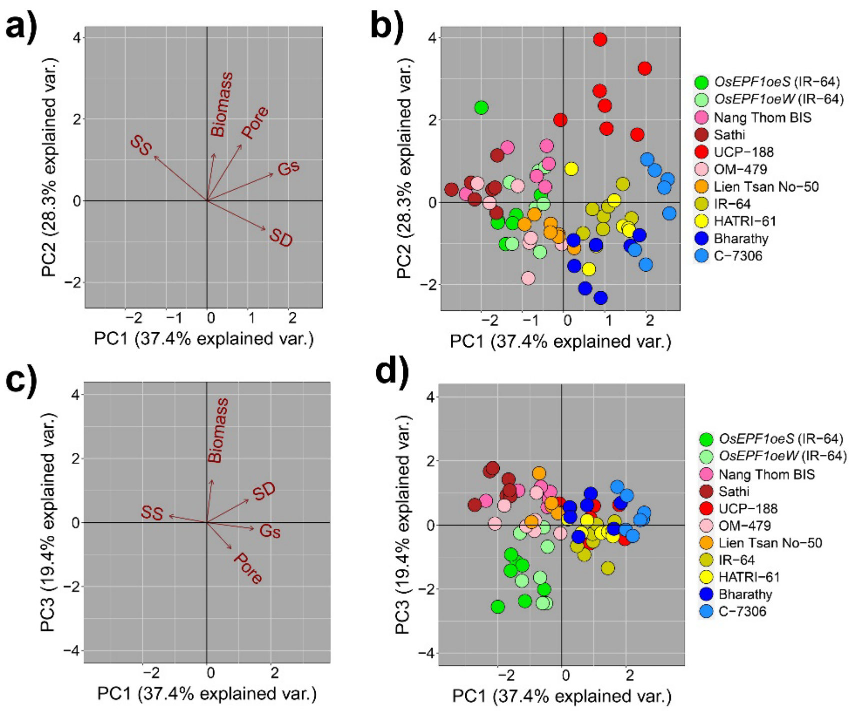

Fig. S3. Principle component analysis (PCA) of stomatal, biomass and gas exchange measurements conducted on nine selected natural rice (*Oryza sativa* L.) varieties and two transgenic lines. (**a**) PCA biplot of PC1 and PC2 with arrows highlighting the relationship between the variables used. (**b**) Same biplot as in (a) with samples included and arrows removed. The rice varieties separate along PC1 according to SS and SD with UCP-188 forming an individual cluster based on differences associated mainly with pore size and biomass along PC2. (**c**) PCA biplot of PC1 and PC3 with arrows highlighting the relationship between the variables used. (**d**) Same biplot as in (c) with samples included and arrows removed. *OsEPF1oe* plants separate from the other varieties along PC3 again mainly due to differences in pore size and biomass. *n* = 7.

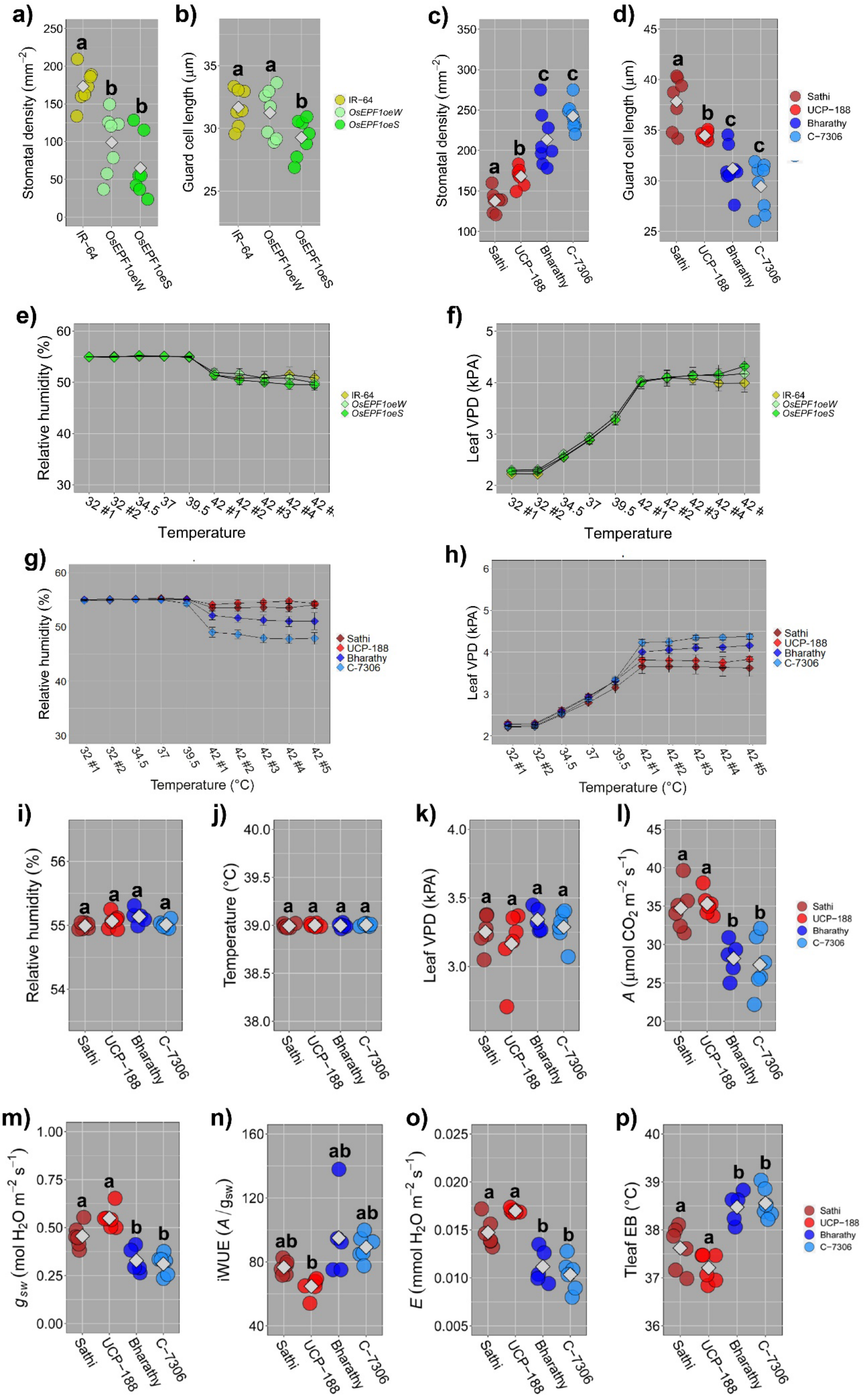

a)
Stomatal density (mm^{-2})
IR-64
OsEPF1oeW
OsEPF1oeS
b)
Guard cell length (µm)
IR-64
OsEPF1oeW
OsEPF1oeS
c)
Stomatal density (mm^{-2})
d)
Guard cell length (µm)
Sathi
UCP-188
Bharathy
C-7306
e)
Relative humidity (%)
Temperature
f)
Leaf VPD (kPA)
Temperature
g)
Relative humidity (%)
Temperature (°C)
h)
Leaf VPD (kPA)
Temperature (°C)
i)
Relative humidity (%)
j)
Temperature (°C)
k)
Leaf VPD (kPA)
l)
A (µmol CO$_2$ m^{-2} s^{-1})
m)
g_{sw} (mol H$_2$O m^{-2} s^{-1})
n)
iWUE (A / g_{sw})
o)
E (mmol H$_2$O m^{-2} s^{-1})
p)
Tleaf EB (°C)

Fig. S4. The impacts of increased temperature and associated vapour pressure deficit (VPD) rises on rice (*Oryza sativa* L.) with differences in stomatal size (SS) and density (SD). SD and SS relating to Fig.6 for (**a**-**b**) IR-64 and *OsEPF1oeW* and *OsEPF1oeS* and (**c**-**d**) Sathi, UCP-188, Bharathy and C-7306. (**e**) Relative humidity (RH) and (**f**) leaf VPD for IR-64 and *OsEPF1oeW* and *OsEPF1oeS*. (**g**) RH and (**h**) leaf VPD for Sathi, UCP-188, Bharathy and C-7306. Fixed RH at 55% and temperature at 39 ˚C experiment comparing Sathi, UCP-188, Bharathy and C-7306 showing (**i**) RH, (**j**) chamber temperature, (**k**) leaf VPD, (**l**) photosynthesis (*A*), (**m**) stomatal conductance (g_{sw}), (**n**) intrinsic water-use efficiency (A/g_{sw}; iWUE), (**o**) Transpiration (*E*) and (**p**) Tleaf energy balance (EB). Different letters in a-d and e-l indicate a significant difference between the means (One-way ANOVA, Tukey HSD test, $P < 0.05$). Grey diamonds represent means. a-h, n = 7-8 plants. i-p, n = 5-6 plants.
